# Antiinflammatory effects of cucurbitacins and sorafenib in Hepg2 cells by modulating the IκB/NF-κB/COX-2 pathway through Akt signaling

**DOI:** 10.55730/1300-0152.2747

**Published:** 2025-01-15

**Authors:** Muhammed Mehdi ÜREMİŞ, Yusuf TÜRKÖZ, Nuray ÜREMİŞ

**Affiliations:** Department of Medical Biochemistry, Faculty of Medicine, İnönü University, Malatya, Turkiye

**Keywords:** Cucurbitacin, sorafenib, HepG2, inflammation, NFκB, COX-2

## Abstract

**Aim:**

Cucurbitacins possess antitumor, antiproliferative, and antiinflammatory properties. This study aims to examine the antiinflammatory effects of CuD, CuI, and CuE on NF-κB, iNOS, COX-2, and Akt and compare their cytotoxic and antiinflammatory effects on HepG2 cells with sorafenib, the primary chemotherapeutic agent used in HCC treatment.

**Methods:**

Cytotoxic effects of cucurbitacins and sorafenib on HepG2 cells were evaluated using MTT and LDH assays, along with Annexin V, MMP, and comet assays. Levels of proteins related to the Akt/NF-κB pathway and COX-2, iNOS, and NO were also measured.

**Results:**

Our study showed that CuD, CuI, CuE, and sorafenib have antiproliferative and cytotoxic effects on HepG2 cells. Cucurbitacins induced apoptosis at lower concentrations than sorafenib and, like sorafenib, reduced p-Akt, p-IκBα protein levels, and nuclear translocation of NF-κB in a dose-dependent manner. Moreover, all compounds significantly decreased COX-2, iNOS, and NO levels, especially at 5 μM concentration.

**Conclusion:**

These results indicate that cucurbitacins exert antiinflammatory effects on HepG2 cells by modulating the PI3K/Akt/NFκB signaling pathway, thereby reducing COX-2, iNOS, and NO levels. These effects are similar to those of sorafenib.

## 1. Introduction

Although cancer remains a significant cause of global mortality, the incidence and fatality rates of different types of cancer are consistently rising each year (Bray et al., 2018; Uremis et al., 2022b). Various factors, such as infection, obesity, exposure to ultraviolet radiation, smoking, alcohol consumption, and genetic predisposition, are associated with the development of cancer ( Uremis et al., 2017b; Rider et al., 2021). These factors lead to the overactivation of multiple signaling pathways that regulate cellular survival, chromosome and DNA mutation, gene expression, cell cycle proliferation, and the disruption of autophagic cell death in cancer cells (Hanahan and Weinberg, 2011; Ceylan et al., 2020).

The nuclear factor kappa B (NF-κB) and PI3K/Akt pathways interact in complex ways, resulting in decreased survival rates, increased proliferation, and migration in cancer cells (Burow et al., 2000; Annunziata et al., 2010; Ahmad et al., 2013). The catalytic subunits of PI3K can directly activate NF-κB or indirectly activate it through the phosphorylation of the IκB kinase (IKK) complex (Ozes et al., 1999; Gilmore, 2006; Ghoneum et al., 2020). Cytokines, hormones, and various growth factors activate PI3K, leading to the phosphorylation of Akt. Phosphorylated Akt (p-Akt) then facilitates the localization of NF-κB to the nucleus by inducing IκBα phosphorylation (Yu et al., 2017; Uremis et al., 2023). This underscores a direct relationship between the transcription of PI3K, Akt, and NF-κB subunits.

NF-κB is a nuclear transcription factor sensitive to redox and regulates gene expression for cell survival, growth, and maturation in various biological processes (Thoma and Lightfoot, 2018). As a redox-sensitive transcription factor, NF-κB is modulated by free radicals and reactive oxygen species formed in mitochondria (Morgan and Liu, 2011). In normal human cells, NF-κB activation is crucial for normal tissue development and homeostasis in various systems, including the immune, nervous, and hepatic systems (Dutta et al., 2006). However, transcription factors, cytokines, ultraviolet radiation, free radicals, oxidized LDL, and bacterial antigens can activate NF-κB (Mitchell et al., 2016). Consequently, NF-κB exhibits effects such as blocking apoptosis, stimulating cell proliferation, increasing inflammation, and inducing angiogenesis in cancer cells (Dolcet et al., 2005; Luqman and Pezzuto, 2010).

COX-2, an isoform of cyclooxygenases (COX), is an effector enzyme that can be activated by NF-κB (Lee et al., 2007). It is known that COX-2 stimulation induces cell proliferation, inhibits apoptosis, and increases cell migration, adhesion, and invasion in cancer cells (Ye et al., 2020). Increased levels of intracellular COX-2 prevent programmed cell death by decreasing arachidonic acid and prostaglandins in the cyclooxygenase pathway and inhibiting lipid peroxidation (Cao and Prescott, 2002). Additionally, there is an interplay between nitric oxide (NO) and prostaglandin reaction systems. NO can activate COX-2 (Mollace et al., 2005). Prostaglandin metabolites produced by activated COX-2 inhibit apoptosis by blocking the NO signaling pathway (Williams et al., 2003; Mollace et al., 2005).

For centuries, natural plants have been utilized in traditional medicinal practices, particularly in Asian societies, to prevent and treat various ailments (Shah et al., 2014). Extracts and purified compounds derived from these plants are used as source materials in drug development to improve health (Dhouibi et al., 2020). Cucurbitacins, a group of phytochemicals, have been isolated from various plant families and are classified alphabetically based on the positions of functional groups and their chemical properties (Kaushik et al., 2015). In vitro studies have examined the free radical scavenging (Tannin-Spitz et al., 2007) and analgesic (Jayaprakasam et al., 2003; Recio et al., 2004), antiproliferative (Sikander et al., 2019), and antitumorigenic (Sikander et al., 2019) effects of cucurbitacins in different cell lines, as well as in vivo in animal models. It is also known that the antiinflammatory properties of cucurbitacins play a decisive role in their hepatoprotective effects (Bartalis and Halaweish, 2011). The protective potential of cucurbitacin D and its derivatives against inflammation-related liver toxicity has been demonstrated by suppression of TNF-α, IL-6, and inhibition of NF-κB phosphorylation in lipopolysaccharide (LPS)-stimulated hepatic stellate cells (Arjaibi et al., 2017).

Although not of liver origin, L929 cells derived from mouse connective tissue are widely used in cytotoxicity studies to evaluate the effects of therapeutic compounds on non-cancerous cells (Zange and Kissel, 1997). In this study, both hepatocellular carcinoma (HCC) cells and L929 fibroblast cells were used as controls to evaluate the apoptotic, antiinflammatory, and selective toxicities of cucurbitacins isolated from the Ecballium elaterium plant. However, few studies have evaluated cucurbitacins’ apoptotic and antiinflammatory effects together. Therefore, our study aimed to investigate the modulatory effect of cucurbitacins D, I, and E, isolated from the *Ecballium elaterium* plant, on the PI3K/Akt/NF-κB pathway and to assess their potential antiinflammatory and cytotoxic effects by comparing them with sorafenib, a drug used in the treatment of HCC.

## 2. Methods

### 2.1. Preparation of cucurbitacins

Cucurbitacin D (CuD), cucurbitacin I (CuI), and cucurbitacin E (CuE) were prepared according to previously established methods by our research group (Uremis et al., 2022a; Uremis et al., 2022b; Uremis et al., 2023). In brief, the juice from the *Ecballium elaterium* plant’s fruit was obtained and dried through lyophilization. The resulting lyophilized juice was then subjected to sequential extraction with hexane and chloroform solvents. The components present in the chloroform phase were further purified using high-performance liquid chromatography (HPLC, Shimadzu, LC-10AD VP) to isolate CuD, CuI, and CuE. The obtained cucurbitacins were subsequently collected using a fraction collector and subjected to lyophilization.

### 2.2. Cell culture

The HepG2 Hepatocellular carcinoma cells (HB-8065, ATCC) were cultured following the earlier protocol (Uremis et al., 2022a). HepG2 cells were cultured in a low glucose medium (MEM-α, Gibco, Cat: 51200-046) without nucleosides as well as L929 cells were cultured in Dulbecco’s modified Eagle medium (DMEM high glucose, Gibco, Cat: 41966-029). Both cell lines were cultured and supplemented with 1% penicillin-streptomycin (Gibco, Cat: 10378016) and 10% Fetal Bovine Serum (Gibco, Heat Inactivated, Cat: 10100147). Cells were incubated at 37 °C in the presence of 5% CO2. Cells controlled under an inverted microscope (Leica, DMIL LED) were passaged when they were approximately 70%–80% confluent. Untreated controls received equivalent amounts of DMSO (Sigma, D2650) at the highest CuD, CuI, and CuE concentrations. The maximum concentration of DMSO was maintained below 0.1% v/v. Cell counting was performed at all cell seeding stages (DeNovix CellDrop BF).

### 2.3. MTT assay

The MTT assay was conducted to evaluate cellular viability (Kumar et al., 2018). HepG2 cells were seeded in 96-well plates at 10,000 cells per well and incubated to confluency. In addition, to investigate the effect of cucurbitacin and sorafenib on a normal fibroblast cell line (L929, CRL-6364, ATCC), cells were seeded in 96-well plates at 10,000 cells per well and incubated until confluent. Subsequently, cells were cultured for 24 h with varying concentrations of CuD, CuI, CuE, and sorafenib (0.1–10 μM). 10 μL of MTT (Sigma, M2128) reagents were added at a 0.5 mg/mL concentration in each well. After incubating the plates for 4 h, the wells were decanted, and 100 μL DMSO was added to dissolve the formazan crystals. At the end of this period, the absorbance value of the wells was determined at 570 and 650 nm wavelengths using a microplate reader (Biotek Synergy H1). Three independent experiments were performed to obtain the data.

### 2.4. Cytotoxicity assay

The LDH Kit (Roche, Cytotoxicity Detection Kit^PLUS^, Ref: 04744926001) was utilized to measure cytotoxicity. This assay assesses the release of the intracellular enzyme LDH from cells that have incurred damage. Cells were cultivated in a 96-well plate and incubated for 24 h to facilitate attachment. After the incubation period, different concentrations (0.1–10 μM) of CuD, CuI, and CuE were added to each well and incubated for 24, 48, and 72 h. After adding cucurbitacins, 5 μL of lysis solution was introduced to the positive control (high control), and the plate was subjected to incubation at 37 °C for 15 min. Subsequently, 100 μL of a mixture comprising dye and catalyst was added. Lastly, 50 μL of stop solution was introduced, and controls were established on each plate to ascertain the percentage of cytotoxicity. The calculation for cytotoxicity was as follows: Cytotoxicity (%) = [OD^sample^ − OD^Low cont^/OD^High cont^ − OD^Low cont^] × 100. Three independent experiments were performed to obtain the data.

### 2.5. Mitochondrial membrane potential assay (ΔΨM)

The JC1- Mitochondrial Membrane Potential Assay Kit (Abcam, ab113850) was used for this assay. HepG2 cells were seeded in 96-well plates at 10,000 cells per well and incubated to confluency. Subsequently, the cells were exposed to 1 μM of Cucurbitacins and sorafenib and incubated for 48 h. Subsequently, the JC-1 reagent was added to the plates and incubated. Finally, fluorescent images of the plate were captured using a fluorescent microscope (Zeiss, Axio Vert.A1) with rhodamine (540/570 nm excitation/emission) or Texas red (590/610 nm excitation/emission) as the fluorophores and ImageJ software (Version 1.53e, National Institutes of Health, USA) was used for quantitative analysis.

### 2.6. Comet assay

HepG2 cells were initially seeded into flasks and incubated for 24 h. Once the cells reached confluence, Cucurbitacins and sorafenib were included in the cells at a dose of 5 μM and incubated for 48 h. The hepatocarcinoma cells were then harvested from the culture and precipitated through centrifugation at 130 × g for 5 min. Subsequently, 2 mL of PBS (Phosphate-buffered saline) was added to the resulting pellet. From this mixture, 80 μL was extracted and combined with 280 μL of low melting point agarose (LMA, amresco, Agarose II, Ref: 0815) in a 37 °C water bath. 75 μL of the LMA-cell mixture was pipetted onto slides covered with melt agar (Sigma, A0169) and covered with coverslips. The slides were incubated at +4 °C, and the prepared slides were kept in the dark in cold lysis solution for 1 h at +4 °C. The lysed slides were positioned in the electrophoresis tank for 20 min in the running buffer. Following 25 min of electrophoresis under a 300-mA current, the slides were transferred to neutralization liquid for 15 min in the dark. Finally, the slides were washed with cold dH_2_O, dried, and treated with 1 μg/mL DAPI (Sigma, D9542) dye. The slides were then covered with a coverslip, and images were taken. All the data were analyzed for tail length and olive moment by OpenComet v1.3.1 using ImageJ version 1.53. Three independent experiments were performed to obtain the data.

### 2.7. Apoptosis assay

Apoptosis analysis followed the protocols described in our previous research (Uremis et al., 2022a; Uremis et al., 2022b). FITC Annexin V Apoptosis Detection Kit I (BD Pharmingen, Cat: 556547) was used for this assay. HepG2 cells were seeded in 6-well plates at a density of 4 × 10^5^ cells per well and incubated for 24h at 37 °C. Cells were treated with different concentrations (1 and 5 μM) of cucurbitacin at 3.5 mL/well media volume. After a 24-h incubation period, cells were trypsinized (Gibco, Trypsin-EDTA, 0.25%, Cat: 25200072) and centrifuged, and the pellet was collected. The cells were then washed with PBS. The pellets were suspended in 1 mL of 1X Annexin V binding buffer, and 100 μL of this suspension was labeled with 5 μL FITC Annexin V and 5 μl PI. After labeling, the suspension was incubated for 15 min, and 400 μL of 1X Annexin V binding buffer was added. Analyses were performed within 15 min with a flow cytometer (Navios EX, Beckman Coulter Inc., Brea, CA, USA). Kaluza Analysis software (Beckman Coulter, USA) was used for data analysis.

### 2.8. Nuclear fractionation

The nuclear fraction of the cell was acquired using the methodology outlined previously by Senichkin et al. (Senichkin et al., 2021). To prepare the cells, cells were first seeded in 6-well plates and confluent cells were trypsinized followed by centrifugation. Subsequently, the resulting cell pellet was suspended in a hypotonic solution comprising 0.1% NP-40 and kept under refrigeration. Following this, homogenization of the cells was performed using a cell grinder (Isolab, Tissue grinder, I.078.19.001.001). The resulting homogenate was centrifuged at 1000 × g for 5 min at a temperature of +4 °C. An isotonic solution containing 0.1%–0.3% NP-40 was then introduced to the cell pellet, and further homogenization (BioSpec, MiniBeadBeateer-16, USA) of the cells was carried out. The resulting homogenate was maintained on ice for 5–10 min and then centrifuged. The supernatant, which constitutes the nuclear fraction, is stored at −80 °C.

### 2.9. Protein analysis

HepG2 cells were cultured in six wells and exposed to varying concentrations (0.5, 1, and 5 μM) of Cucurbitacins and sorafenib (1, 5, and 10 μM) for 48 h. The media was removed, and the cells were washed with cold PBS and then incubated in RIPA lysis buffer for 20–30 min on ice and harvested; centrifugation was then carried out at 20,000 × g at 4 °C for 10 min. Protein levels of the lysate were measured, 30 μg of proteins were loaded in 10% SDS-PAGE, and then proteins were transferred to PVDF membranes (Serva, 0.45 μm). The membrane was blocked in 5% nonfat dry milk (Serva, Skim milk powder, Cat: 42590) for 1h at room temperature. The blocked membrane was incubated with primary antibodies overnight at 4 °C: (Akt [Cat: 4691], p-Akt [Cat: 4060], IκBα [Cat: 4814], p-IκBα [Cat: 2859], NF-κB [Cat: 8242], and β-actin [Cat: 4970]) were provided by Cell Signaling Technology. After incubating the membranes with primary antibodies, the corresponding secondary antibodies conjugated with HRP (Cat: 7076 and 7074) were added and allowed to react for one hour at room temperature. Following this, blots were imaged with ECL substrate (Pierce ECL Plus Western Blotting Substrate, Thermo Scientific, Cat: 32132). Bands were detected with MicroChemi (DNR Bio-Imaging System), and ImageJ software (Version 1.53e, National Institutes of Health) was used for band quantitative densitometric analysis. Three independent experiments were performed to obtain the data.

### 2.10. NO, COX-2, and iNOS measurement

HepG2 cells were subjected to various concentrations of purified cucurbitacins (0.5, 1, and 5 μM) and sorafenib (1, 5, and 10 μM) for 48 h. After incubation, the medium was aspirated and substituted with phosphate buffer (pH 7.4). Subsequently, the cells were scraped and lysed. The resulting mixture was centrifuged to obtain the supernatant. The supernatant was then incubated at a temperature of +4 °C for subsequent analysis. The Griess reaction determined the nitrite content within the supernatant, and the recorded value was regarded as the total nitrite content. The measurement technique utilized in this study was the methodology presented by Özbek et al. (Ozbek et al., 2000). To elaborate, the supernatant was initially deproteinized using ZnSO_4_ and NaOH. A NO reagent containing nitrate reductase was prepared, added to the deproteinized samples, and incubated. LDH reagent was prepared and added to the samples. Following an additional incubation period of 30 min at 37 °C, Griess reagent was added, and measurements were carried out. The evaluation of COX-2 and iNOS was conducted employing the ELISA kit, adhering to the manufacturer’s protocol (SunRed). We used an ELISA microplate reader (BioTek Synergy H1, BioTek Instruments) and a data analysis program (Gen5, BioTek Instruments). Three independent experiments were performed to obtain the data.

### 2.11. Statistical analysis

All analysis was conducted using GraphPad Prism. The Shapiro-Wilk test was used for normality distribution test. Group comparisons were performed using a two-way analysis of variance (ANOVA). Kruskal-Wallis was used when the data did not follow a normal distribution.

## 3. Results

### 3.1. Effects of cucurbitacins and sorafenib on HepG2 proliferation

**Figure 1:**
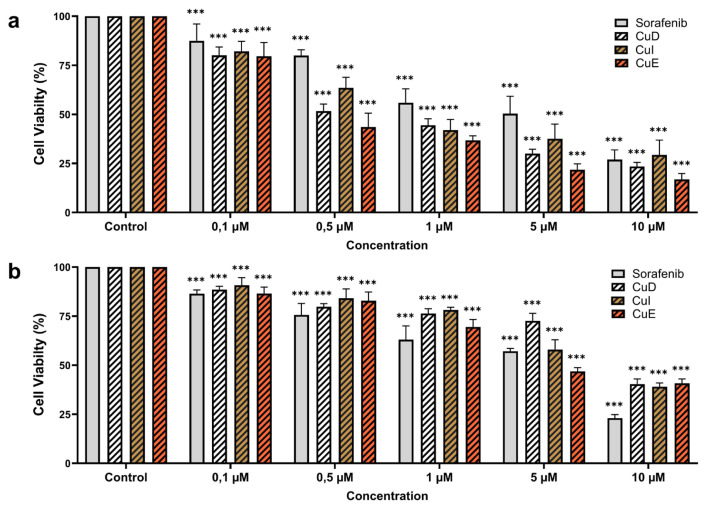
CuD, CuI, CuE, and sorafenib exhibit an antiproliferative effect on HepG2 and L929 cell lines. The MTT assay was utilized to evaluate cell viability. HepG2 **(a)** and L929 **(b)** cell lines were subjected to varying concentrations of cucurbitacins and sorafenib for 48 h. *p < 0.05, **p < 0.01, and ***p < 0.001 indicating significant differences compared to the control, and “ns” indicating no significant difference.

The MTT assay evaluated the effects of cucurbitacins on the viability of HepG2 (Figure 1a) and L929 (Figure 1b) cell lines. The antiproliferative properties of cucurbitacins and sorafenib were investigated at concentrations of 0.1, 0.5, 1, 5, and 10 μM by subjecting the HepG2 and L929 cells to these compounds for 48 h. The results revealed that cucurbitacins and sorafenib exhibited a decrease in cell viability. Furthermore, when comparing the impact of cucurbitacins on cell proliferation with sorafenib, it was observed that lower concentrations of cucurbitacins inhibited the proliferation of HepG2 cells. The calculated IC_50_ concentrations of CuD, CuI, CuE, and sorafenib in the HepG2 cell line were 0.306, 0.344, 0.279, and 0.956 μM, respectively. Similarly, the IC_50_ concentrations of CuD, CuI, CuE, and sorafenib calculated in the L929 cell line were 1.632, 1.016, 1.444, and 1.504 μM, respectively (Figure 1a, Figure 1b).

### 3.2. LDH cytotoxicity assay

**Figure 2:**
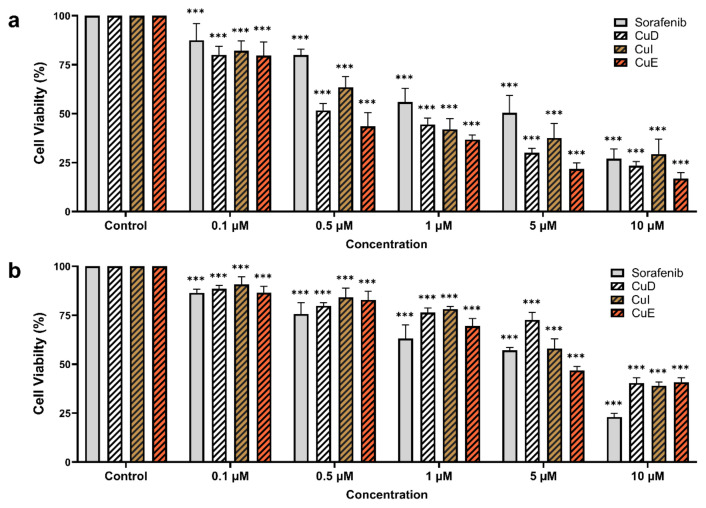
LDH release from HepG2 cells treated with cucurbitacins and sorafenib. HepG2 cells were treated with various concentrations of CuD **(a)**, CuI **(b)**, CuE **(c)**, and sorafenib **(d)** for 24h, 48h, and 72h. The lactate dehydrogenase (LDH) leakage (% of total LDH) was measured. *p < 0.05, **p < 0.01, and ***p < 0.001 indicating significant differences compared to the control, and “ns” indicating no significant difference.

The LDH Assay kit (provided by Abcam) was utilized to ascertain the cytotoxicity of HepG2 cells, which had their membrane integrity compromised by applying CuD (Figure 2a), CuI (Figure 2b), CuE (Figure 2c), and sorafenib (Figure 2d). Varied concentrations of cucurbitacins were administered over 24, 48, and 72 h to evaluate the LDH activity. The cytotoxic impact induced by cucurbitacins was compared to that of the control group. Cytotoxicity exhibited an escalation in dosage and time for different CuD, CuI, CuE, and sorafenib concentrations. The most prominent LDH activity was observed at concentrations of 5 and 10 μM cucurbitacins.

### 3.3. Mitochondrial membrane potential (MMP, ΔΨm)

**Figure 3:**
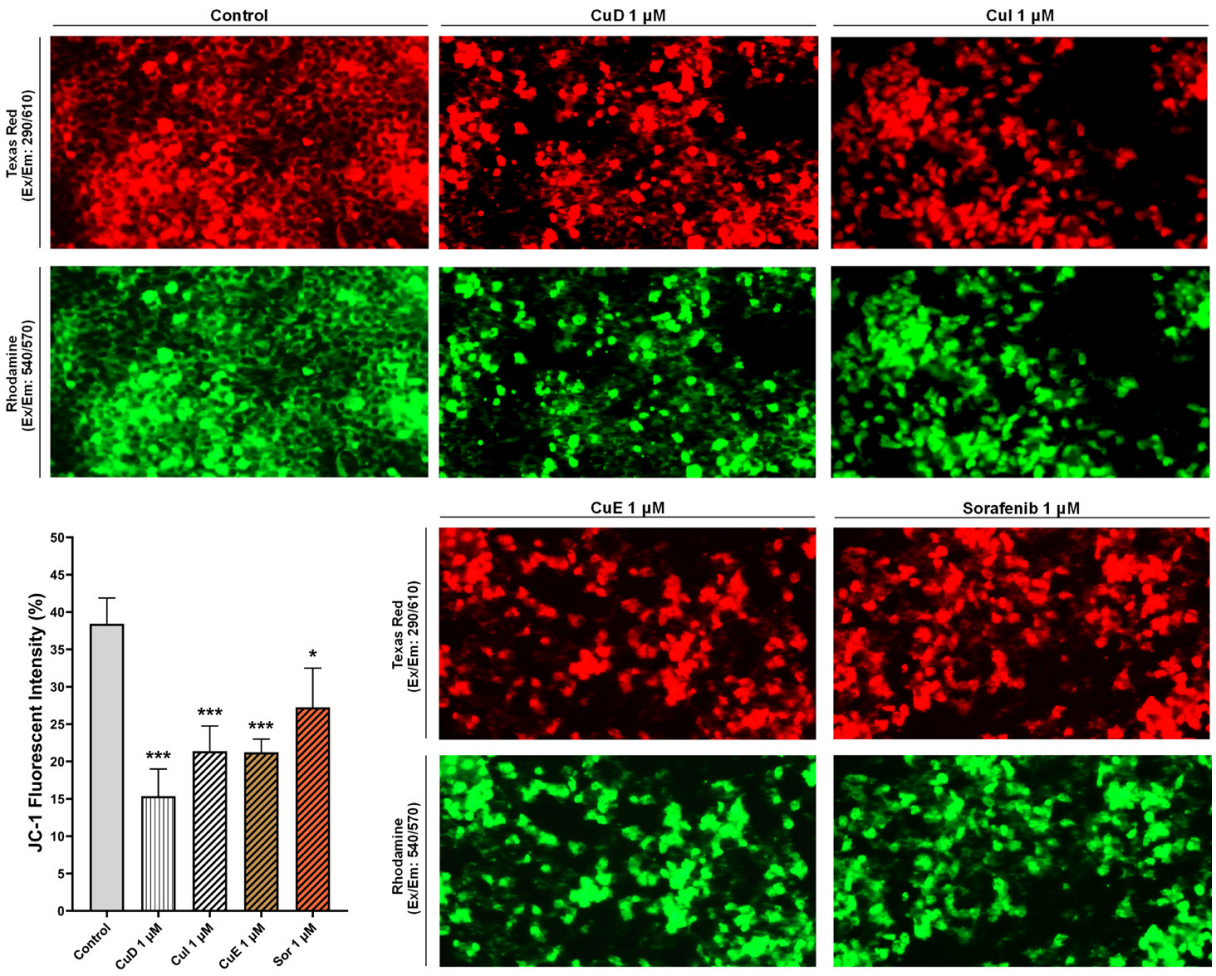
Effects of cucurbitacins and sorafenib on mitochondrial membrane potential (MMP) in HepG2 cells. Representative fluorescence microscopy images of HepG2 cells stained with JC-1, indicating MMP changes. Texas Red fluorescence represents intact mitochondria with high membrane potential. Cells were treated with CuD (1 μM), CuI (1 μM), CuE (1 μM), and sorafenib (1 μM) for 48 h. The bar graph quantifies the percentage of JC-1 fluorescence intensity for stained regions, determined using ImageJ software. Data reflect the proportion of stained areas (%), highlighting the significant effects of all treatments. *p < 0.05, **p < 0.01, and ***p < 0.001 indicating significant differences compared to the control.

The fluorescence microscopy technique using the cationic dye JC-1 was employed to investigate the mitochondrial membrane potential. The membrane of mitochondria, where oxidative phosphorylation occurs, is closely associated with apoptosis. A distinct red fluorescent dye in the mitochondria indicates active aerobic respiration and reflects the membrane potential. Moreover, a cytosolic green fluorescence can be observed depending on the potential. To investigate MMP, we utilized JC-1 staining to quantify the fluorescence signals in apoptotic or necrotic cells. Initially, HepG2 cells showed a robust red fluorescence, meaning a substantial buildup of JC-1 dye within the mitochondria. Nevertheless, subsequent administration of Cucurbitacins and sorafenib reduced JC-1 dye accumulation and red fluorescence intensity. These findings imply that these compounds diminish mitochondrial membrane potential and facilitate apoptosis (Figure 3).

### 3.4. Effect of cucurbitacins and sorafenib on DNA damage

The comet assay was employed to assess the impact of cucurbitacins and sorafenib on the levels of DNA damage in HepG2 cells. To perform this, a dose of 1 μM of the compounds (close to their IC50 values) was compared to the comet scores of the control group. Subsequent alkaline single-cell electrophoresis demonstrated that the cells in the control group displayed an intact and compact structure. Conversely, administration of a 1 μM concentration of cucurbitacins and sorafenib resulted in the disruption of the cell structure and the formation of a tail, indicating DNA fragmentation. Upon assessing the comet scores, it was observed that the comet tail lengths of CuD, CuI, and CuE were higher than those of the untreated cells, measuring 43.6, 40.01, and 32.3, respectively. Similarly, sorafenib demonstrated a tail length of 31.

**Figure 4:**
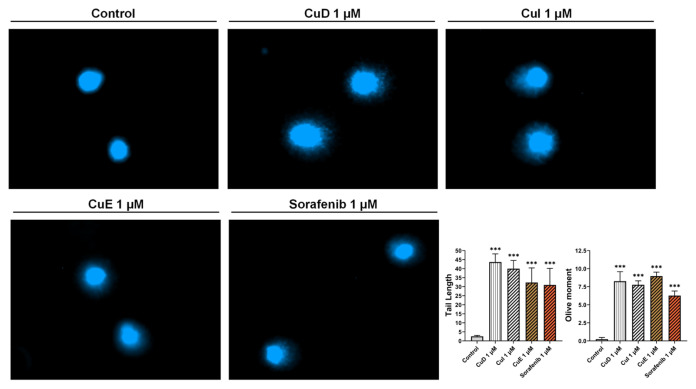
Effect of cucurbitacins and sorafenib on DNA damage. Cucurbitacins and sorafenib induced DNA damage in HepG2 cells. Alkaline gel electrophoresis was used to detect comet images of DNA fragments. After 48 h of incubation with 1 μM of cucurbitacins and sorafenib, HepG2 cells were observed for comet-positive cells under a fluorescent microscope. The levels of DNA damage were determined using the olive moment and tail length. *p < 0.05, **p < 0.01, and ***p < 0.001 indicating significant differences compared to the control, and “ns” indicating no significant difference.

The Olive moment values for CuD, CuI, CuE, and sorafenib were determined to be 8.26, 7.76, 8.96, and 6.26, respectively. These findings suggest that cucurbitacins and sorafenib contribute to increased comet tail length. Furthermore, they imply that cucurbitacins induce more extensive DNA damage than sorafenib at the same concentration (Figure 4).

### 3.5. Effect of cucurbitacins and sorafenib on apoptosis

**Figure 5:**
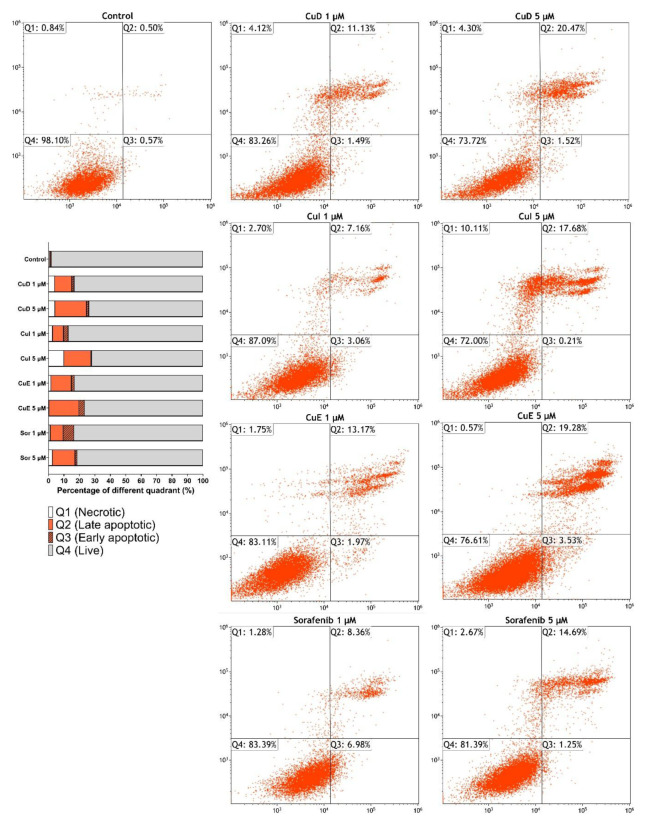
Investigation of the effect of cucurbitacins and sorafenib on the HepG2 cell line using Annexin V flow cytometry. Cucurbitacins and sorafenib induce apoptosis in HepG2 cells at different concentrations. After 48 h of treatment with cucurbitacins and sorafenib, apoptosis was assessed using Annexin V assay. The percentage of Annexin V+/PI+ quadrants in the upper right measured the extent of apoptosis. The 5 μM groups showed a significantly higher percentage of apoptosis than the 1 μM groups.

Annexin V was employed to ascertain the apoptotic impact of cucurbitacins and sorafenib in HepG2 cells. It was ascertained that both cucurbitacins and sorafenib exhibited a cytotoxic effect in contrast to the control group across both dosages. An escalation in the apoptotic populace was observed concomitant with the increase in dosage. Upon evaluating concentrations of 5 μM, late apoptosis rates in HepG2 cells were determined as 20.47% for CuD, 17.68% for CuI, 19.28% for CuE, and 14.69% for sorafenib. At the same concentration, Cucurbitacins were discovered to possess a more significant number of apoptotic cells than sorafenib (Figure 5).

### 3.6. Effect of cucurbitacins and sorafenib on Akt, NF‐κB, IκBα proteins

**Figure 6:**
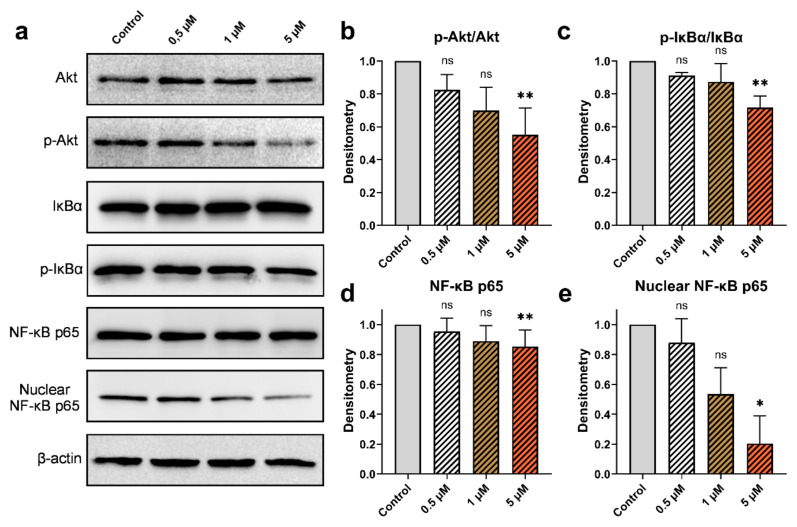
Effect of CuD on p-Akt/Akt, p-IκBα/IκBα, and NF-κB protein expression. (a) Western blot analysis of total Akt, phosphorylated Akt (p-Akt), IκBα, phosphorylated IκBα (p-IκBα), NF-κB p65, and nuclear NF-κB p65 levels in HepG2 cells treated with cucurbitacin D at concentrations of 0.5, 1, and 5 μM for 48 h (b–e) Densitometric analysis of p-Akt/Akt, p-IκBα/IκBα, total NF-κB p65, and nuclear NF-κB p65 levels, respectively. Data are expressed as mean ± SD (n = 3). Statistical analysis was performed using Kruskal-Wallis, with *p < 0.05, **p < 0.01, and ***p < 0.001 indicating significant differences compared to the control, and “ns” indicating no significant difference.

**Figure 7:**
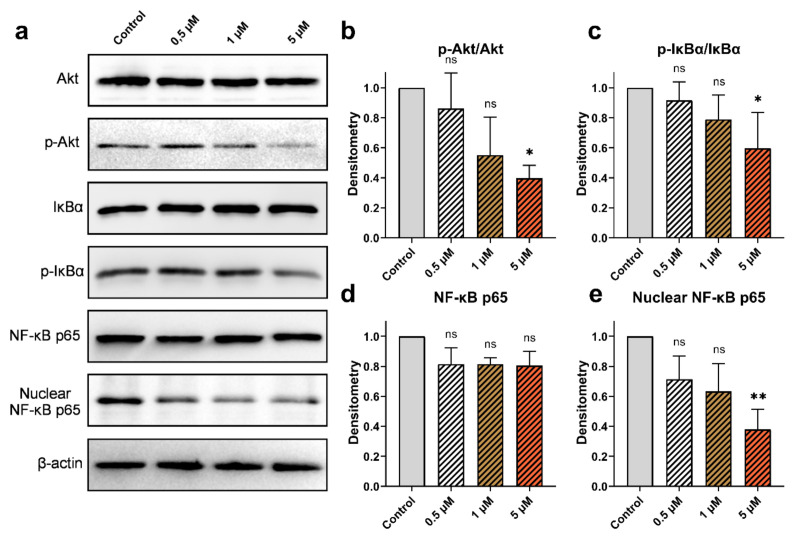
Effect of CuI on p-Akt/Akt, p-IκBα/IκBα, and NF-κB protein expression. (a) Western blot analysis of total Akt, phosphorylated Akt (p-Akt), IκBα, phosphorylated IκBα (p-IκBα), NF-κB p65, and nuclear NF-κB p65 levels in HepG2 cells treated with cucurbitacin I at concentrations of 0.5, 1, and 5 μM for 48 h(b–e) Densitometric analysis of p-Akt/Akt, p-IκBα/IκBα, total NF-κB p65, and nuclear NF-κB p65 levels, respectively. Data are expressed as mean ± SD (n = 3). Statistical analysis was performed using Kruskal-Wallis, with *p < 0.05, **p < 0.01, and ***p < 0.001 indicating significant differences compared to the control, and “ns” indicating no significant difference.

**Figure 8:**
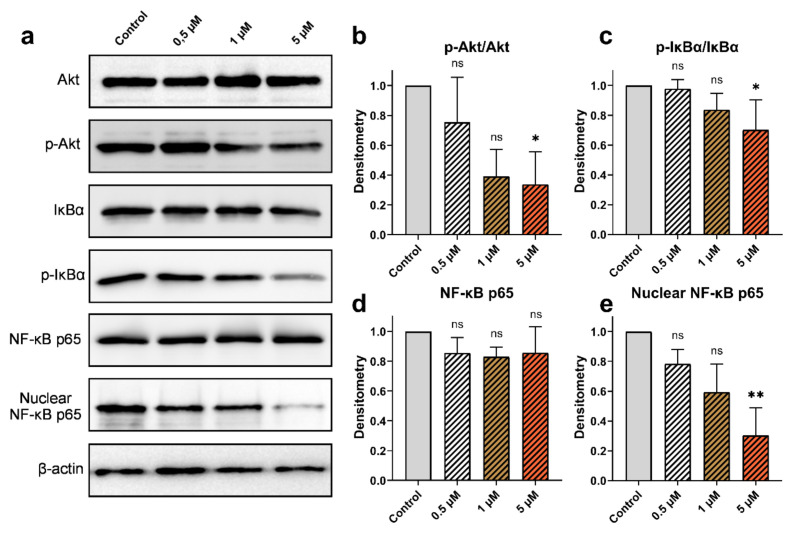
Effect of CuE on p-Akt/Akt, p-IκBα/IκBα, and NF-κB protein expression. (a) Western blot analysis of total Akt, phosphorylated Akt (p-Akt), IκBα, phosphorylated IκBα (p-IκBα), NF-κB p65, and nuclear NF-κB p65 levels in HepG2 cells treated with cucurbitacin E at concentrations of 0.5, 1, and 5 μM for 48 h (b–e) Densitometric analysis of p-Akt/Akt, p-IκBα/IκBα, total NF-κB p65, and nuclear NF-κB p65 levels, respectively. Data are expressed as mean ± SD (n = 3). Statistical analysis was performed using Kruskal-Wallis, with *p < 0.05, **p < 0.01, and ***p < 0.001 indicating significant differences compared to the control, and “ns” indicating no significant difference.

**Figure 9:**
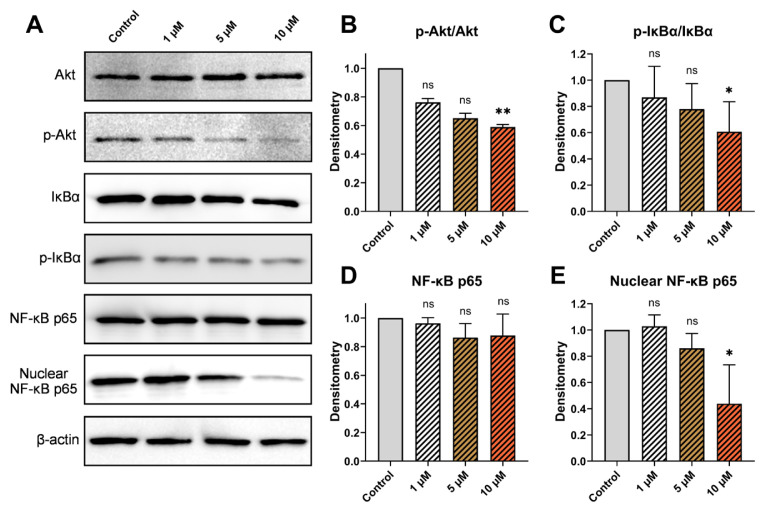
Effect of sorafenib on p-Akt/Akt, p-IκBα/IκBα, and NF-κB protein expression. (a) Western blot analysis of total Akt, phosphorylated Akt (p-Akt), IκBα, phosphorylated IκBα (p-IκBα), NF-κB p65, and nuclear NF-κB p65 levels in HepG2 cells treated with sorafenib at concentrations of 0.5, 1, and 5 μM for 48 h (b–e) Densitometric analysis of p-Akt/Akt, p-IκBα/IκBα, total NF-κB p65, and nuclear NF-κB p65 levels, respectively. Data are expressed as mean ± SD (n = 3). Statistical analysis was performed using Kruskal-Wallis, with *p < 0.05, **p < 0.01, and ***p < 0.001 indicating significant differences compared to the control, and “ns” indicating no significant difference.

The impact of Cucurbitacins on the activation of Akt, IκBα, and NF-κB in HepG2 cells was assessed through western blot analysis. No alterations were observed in the levels of Akt protein upon the administration of CuD, CuI, and CuE. However, a decrease dependent on the dose was observed in the levels of its phosphorylated forms. Moreover, p-IκBα protein levels were reduced. The levels of NF-κB in both the overall protein fraction and the nuclear protein fraction, as well as the protein levels of IκBα, were examined to analyze the compounds’ relationship with inflammation. After administering various concentrations of cucurbitacins, a significant downregulation of the nuclear fraction of NF-κB was observed, while the total levels of NF-κB did not exhibit substantial changes. Additionally, the effect of sorafenib on the activation of Akt, IκBα, and NF-κB was investigated. Administered at different doses (1, 5, and 10 μM), sorafenib showed decreased levels of phosphorylated Akt and IκBα proteins, mirroring the effects of cucurbitacins. While sorafenib decreased the level of nuclear NF-κB, it did not cause a significant modification in the total level of NF-κB (Figure 6, Figure 7, Figure 8, Figure 9).

### 3.7. Effect of cucurbitacins and sorafenib on COX-2, iNOS, NO

**Figure 10:**
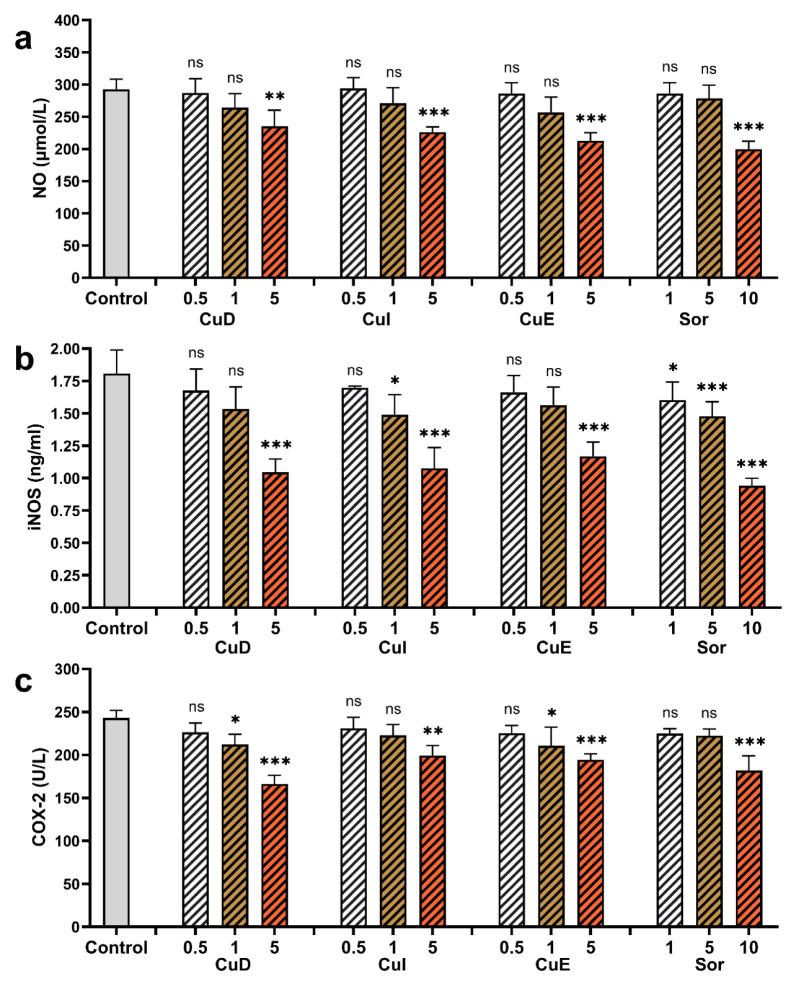
Effect of cucurbitacins and sorafenib on NO, iNOS, and COX-2 levels in HepG2 cells. HepG2 cells were treated with cucurbitacins and sorafenib at concentrations of 0.5, 1, and 5 μM for 48 h, and the levels of NO **(a)**, iNOS **(b)**, and COX-2 **(c)** were measured. *p < 0.05, **p < 0.01, and ***p < 0.001 indicating significant differences compared to the control, and “ns” indicating no significant difference.

There is a direct correlation between inflammation and carcinogenesis. NF-κB, which is excessively activated in carcinogenesis, regulates the COX-2 and iNOS enzymes that have a crucial role in the inflammatory process. It is well established that the activation of NF-κB triggers the activation of the iNOS enzyme, which, in turn, facilitates the production of NO (McAdam et al., 2012). Therefore, we also explored the impact of cucurbitacins and sorafenib on nitrite accumulation. Treating HepG2 cells with a concentration of 5 μM of CuD, CuI, and CuE significantly reduced NO production compared to the control. Furthermore, administering sorafenib at a concentration of 10 μM resulted in a significant decrease in nitrite production compared to the control (Figure 10a). Additionally, we examined the levels of COX-2 and iNOS to elucidate the impact of cucurbitacins and sorafenib on inflammation in hepatocellular carcinoma. When administered to HepG2 cells, Cucurbitacins and sorafenib exhibited a dose-dependent inhibition of COX-2 and iNOS (Figure 10b, Figure 10c).

## 4. Discussion

In terms of global mortality and incidence, liver cancer has the fourth-highest mortality rate. Its incidence is increasing yearly, with over 800,000 new cases reported annually (Bray et al., 2018). The primary causes of chronic infections include hepatitis B and C viruses, as well as the consumption of aflatoxin-contaminated food, excessive alcohol consumption, and cirrhosis, leading to liver inflammation and angiogenesis (Dimri and Satyanarayana, 2020; Caliskan et al., 2021). Extensive research has been conducted to explore the chemoprotective properties of compounds derived synthetically or from natural sources to alleviate the burden of cancer on public health. Specifically, there has been a focus on natural compounds that offer high therapeutic efficacy at a low cost, targeting the molecular level to mitigate the impact of viruses and procarcinogens (Rahman et al., 2020).

The fruit of the *Ecballium elaterium* plant is primarily employed in traditional medicine, particularly in Asian countries, for treating rhinosinusitis and jaundice (Eti et al., 2018; Greige-Gerges et al., 2007). Numerous studies have investigated the therapeutic effects of cucurbitacins, which are present in this plant, against various diseases. These compounds have demonstrated antitumor, antiinflammatory, antimicrobial, and hepatoprotective properties (El Naggar el et al., 2015; Arslan et al., 2016; Jafargholizadeh et al., 2016; Jafargholizadeh et al., 2018). In this study, we sought to examine the effects and molecular mechanisms of CuD, CuI, and CuE, extracted from the *Ecballium elaterium* plant, on cytotoxicity, apoptosis, and inflammation in the hepatocellular carcinoma HepG2 cell line. Additionally, we compared the potential antiinflammatory mechanism of these compounds with that of sorafenib, a chemotherapeutic agent commonly used to treat hepatocellular carcinoma.

One of the in vitro tests commonly used to assess the toxic properties of pharmaceutical compounds is the LDH cytotoxicity test (Riss and Moravec, 2004). The LDH enzyme plays a crucial role in indicating the flow direction in aerobic and anaerobic glycolysis, cellular metabolic status, and the impact of malignant transformation on biochemical processes (Chen et al., 2019). Previous investigations have shown that applying CuE to human prostate cancer cells produces a dose-dependent cytotoxic effect (He et al., 2017). Similarly, CuI applied to lung cancer cells induced significant cytotoxicity (Zhu et al., 2018). Our cytotoxicity results demonstrate that administration of HepG2 cells with various concentrations of cucurbitacins for 24, 48, and 72 h caused both a dose- and time-dependent increase in LDH leakage, consistent with the findings from the MTT assay. Specifically, at the highest concentration (10 μM), cucurbitacins notably induced membrane damage, leading to subsequent LDH leakage.

The literature reveals several in vitro studies demonstrating cucurbitacins’ ability to induce apoptosis by causing mitochondrial damage in various types of cancer. For example, Hung et al. showed that CuE causes apoptosis in OSCC cells by increasing the activation of Caspase-3, an apoptotic factor, while significantly reducing mitochondrial membrane potential (Hung et al., 2013). Ishii et al. stated that CuD increases apoptosis-related Caspase-3, Caspase-9, and PARP levels, reduces antiapoptotic Bcl-2 levels, and decreases mitochondrial membrane potential in endometrial and ovarian cancer cells (Ishii et al., 2013). Furthermore, Guo et al. (Guo et al., 2014) discovered that CuB induces DNA damage by inducing G2/M cell cycle arrest in lung cancer cells. Our research examined the effects of Cucurbitacins and sorafenib on apoptosis, MMP, and DNA damage. Flow cytometry analysis demonstrated a dose-dependent increase in the percentage of apoptotic cells when HepG2 cells were exposed to CuD, CuI, CuE, and sorafenib. Furthermore, ΔΨm and alkaline comet assays revealed that these compounds decreased mitochondrial membrane potential and induced DNA damage by promoting DNA fragmentation.

In previous studies conducted by our team, CuD, CuI, and CuE were investigated in detail to regulate apoptosis mechanisms and oncogenic signaling pathways via reactive oxygen species (ROS) in hepatocellular carcinoma cells (Uremis et al., 2022a; Uremis et al., 2022b; Uremis et al., 2023). These studies revealed that cucurbitacins regulate the Bax/Bcl-xL balance among Bcl-2 family proteins, increase caspase activation, and induce apoptosis by increasing intracellular oxidant levels. Our findings are consistent with previous literature showing that cucurbitacins trigger programmed cell death via apoptotic modulators such as caspase cascade, Bax, and Bcl-2 in different cancer cell lines in the 0–1000 nM concentration range (Ma et al., 2016; Sikander et al., 2016; Park et al., 2024). Furthermore, our previous in vitro studies reported that CuD, CuI, and CuE significantly increased the apoptosis rate by suppressing oncogenic signaling pathways (Jak/Stat, PI3K/Akt/mTOR, MAPK/ERK) that show abnormal activity in hepatocellular carcinoma (Uremis et al., 2022a; Uremis et al., 2022b). In addition, it was reported that CuE showed synergistic and apoptotic effects in combination with sorafenib. This combination can be evaluated in new treatment strategies by increasing the efficacy of sorafenib (Uremis et al., 2023). Other studies showing the synergistic effects of cucurbitacins with various chemotherapeutics have similarly shown that combining cucurbitacins and chemotherapeutics may increase cancer cell apoptosis (Kim et al., 2017; Ku et al., 2018).

In this study, unlike our previous studies, the effects of cucurbitacins on the Akt/IκB/NF-κB inflammatory signaling pathway, COX-2, and NO inflammatory markers in hepatocellular carcinoma were evaluated compared with sorafenib. The NF-κB signaling pathway plays an important role in balancing redox homeostasis in cells and the response to inflammation and oxidative stress (Uremis et al., 2022a). The NF-κB signaling pathway is essential for preserving redox homeostasis and responding to cell inflammation and oxidative stress (Thomas et al., 2019). Since NF-κB is considered a regulator of oncogenesis, chemopreventive studies focus on inactivating NF-κB (Capece et al., 2020). Compounds with antitumor potential have been found to induce apoptosis by blocking the activation of NF-κB p65 dimer (Ramyaa et al., 2014; Li et al., 2019; Liu et al., 2020). In a study analyzing the effect of CuD on the IκB/NF-κB signaling pathway in doxorubicin-resistant breast cancer cells, it was found that CuD decreased p-NF-κB levels, thereby inhibiting NF-κB translocation to the nucleus (Ku et al., 2015). In another study, CuE was reported to effectively inhibit the expression of some cytokines, such as TNF-α, and down-regulate the NF-κB signaling pathway. This suggests that CuE may have some potential in regulating adaptive immune function and suppressing the inflammatory response (Xie et al., 2020). Additionally, CuE decreased the expression of IL-1β and TNF-α, as well as suppressed NF-κB nuclear translocation in RAW 264.7 cells (Qiao et al., 2013). Our study demonstrated that CuD, CuI, CuE, and sorafenib significantly down-regulated nuclear NF-κB in HepG2 cancer cells. The results showed that both cucurbitacins and sorafenib induced apoptosis by preventing the translocation of NF-κB to the nucleus.

Cancer is associated with proinflammation, chronic inflammation, and infectious factors (Hussain and Harris, 2007; Uremis et al., 2017a). For example, long-term gastritis is associated with gastric cancer, prostatitis with prostate cancer, inflammatory bowel diseases with colon cancer, and HBV and HBC virus infections with liver cancer (Gan et al., 2013). Inflammation is critical in cancer development by producing ROS and RNS that cause DNA damage. The increased production of proinflammatory mediators such as cytokines, prostaglandins, and NO affects various cancer formation and progression stages, influencing invasion, metastasis, and angiogenesis (Kundu and Surh, 2010). Two essential enzymes, iNOS and COX-2, seem closely related to the link between inflammation and cancer (Morgan and Liu, 2011). Previous studies have showed the antiinflammatory effect of CuB, CuD, CuE, and CuI through the COX-2 enzyme and have reported that cucurbitacins inhibit the COX-2 enzyme (Jayaprakasam et al., 2003). It has also been reported that dihydro cucurbitacin B and CuR mediate the inhibition of inflammation factors such as nitric-oxide synthase and COX-2 (Kaushik et al., 2015). Our study investigated the levels of iNOS and COX-2 proteins to understand better the roles of cucurbitacins and sorafenib in hepatocellular carcinoma. We observed a significant reduction in iNOS and COX-2 protein levels in HepG2 cells treated with different concentrations of cucurbitacins compared to the untreated group.

Similarly, treatment with varying concentrations of sorafenib also led to a decrease in iNOS and COX-2 levels. The inhibitory effect on COX-2 and iNOS was more pronounced with lower concentrations of cucurbitacins than with sorafenib. The decrease in iNOS and COX-2 levels correlated with decreased NF-κB expression. Overall, these findings show that Cucurbitacins and sorafenib suppress proliferation in HepG2 cells by reducing NF-κB, iNOS, and COX-2 protein levels associated with inflammation.

The present study examines the cytotoxic, apoptotic, and antiinflammatory properties of CuD, CuI, and CuE. Furthermore, it seeks to compare the antiinflammatory efficacy of these compounds with sorafenib. Although previous research has demonstrated the antitumor effects of CuD, CuI, and CuE, studies need to investigate the comparative effectiveness of these compounds and sorafenib in hepatocellular carcinoma cells. Hence, our study is the first to reveal that these substances exhibit antiinflammatory effects by inhibiting the NF-κB signaling pathways, COX-2, activation of the iNOS enzyme, and NO production through a mechanism that relies on PI3K/Akt. To obtain a more comprehensive understanding of the inflammation-related molecular mechanisms behind cucurbitacin-induced apoptosis and their chemopreventive potential, further in vivo and in vitro investigations are warranted.
